# Enhanced Network Efficiency of Functional Brain Networks in Primary Insomnia Patients

**DOI:** 10.3389/fpsyt.2018.00046

**Published:** 2018-02-21

**Authors:** Xiaofen Ma, Guihua Jiang, Shishun Fu, Jin Fang, Yunfan Wu, Mengchen Liu, Guang Xu, Tianyue Wang

**Affiliations:** ^1^Department of Medical Imaging, Guangdong Second Provincial General Hospital, Guangzhou, China; ^2^Department of Neurology, Guangdong Second Provincial General Hospital, Guangzhou, China

**Keywords:** primary insomnia, brain network, default mode network, small-world efficiency, resting-state magnetic resonance imaging

## Abstract

Accumulating evidence from neuroimaging studies suggests that primary insomnia (PI) affects interregional neural coordination of multiple interacting functional brain networks. However, a complete understanding of the whole-brain network organization from a system-level perspective in PI is still lacking. To this end, we investigated in topological organization changes in brain functional networks in PI. 36 PI patients and 38 age-, sex-, and education-matched healthy controls were recruited. All participants underwent a series of neuropsychological assessments and resting-state functional magnetic resonance imaging scans. Individual whole-brain functional network were constructed and analyzed using graph theory-based network approaches. There were no significant differences with respect to age, sex, or education between groups (*P* > 0.05). Graph-based analyses revealed that participants with PI had a significantly higher total number of edges (*P* = 0.022), global efficiency (*P* = 0.014), and normalized global efficiency (*P* = 0.002), and a significantly lower normalized local efficiency (*P* = 0.042) compared with controls. Locally, several prefrontal and parietal regions, the superior temporal gyrus, and the thalamus exhibited higher nodal efficiency in participants with PI (*P* < 0.05, false discovery rate corrected). In addition, most of these regions showed increased functional connectivity in PI patients (*P* < 0.05, corrected). Finally, altered network efficiency was correlated with neuropsychological variables of the Epworth Sleepiness Scale and Insomnia Severity Index in patients with PI. PI is associated with abnormal organization of large-scale functional brain networks, which may account for memory and emotional dysfunction in people with PI. These findings provide novel implications for neural substrates associated with PI.

## Introduction

Primary insomnia (PI) is one of the most prevalent chronic sleep disorders. PI refers to difficulty falling asleep or maintaining sleep for at least 1 month. It is associated with sequelae of daytime impairment or clinically significant distress, and it is not attributable to a medical, psychiatric, or environmental cause (1, 2). According to epidemiological reports, 10% of the adult population experiences chronic insomnia, and PI is estimated to occur in 25% of all people with chronic insomnia worldwide (3). Moreover, the rate of PI continues to grow globally with increasing industrialization, urbanization, and work pressures (3). PI results in daytime fatigue, mood disruption, and cognitive impairments, which can lead to various psychiatric and cognitive disorders (e.g., depressive and anxiety disorders) (4, 5). In addition, PI can negatively affect social productivity and life quality, as well as increase accident risk and health-care utilization (6, 7). However, despite the adverse socioeconomic impact of PI, the neurobiological causes and consequences of the disorder are not fully understood.

Recent advances of neuroimaging techniques have provided powerful tools with which to investigate the neurobiological mechanisms of insomnia. To date, many studies have used different neuroimaging modalities to examine insomnia-related alterations in brain structure, function, and metabolism. For example, using structural magnetic resonance imaging (MRI), brain atrophy is consistently observed with PI in a specific set of regions such as the hippocampus (8, 9) and frontoparietal cortex (10, 11). However, functional studies based on functional MRI (fMRI) and positron emission tomography frequently report insomnia-related increases in multiple regions of spontaneous brain activity and metabolism, which may be due to compensatory adaption (12–15). In addition to local alterations, given the interconnected nature of the human brain, an increasing number of studies have begun to examine abnormal interregional functional integration in insomnia. Killgore and colleagues tested the sensory–motor network in patients with sleep dysfunction and found that difficulty in falling asleep was associated with increased functional connectivity between the primary visual cortex and other sensory regions such as the primary auditory cortex, olfactory cortex, and supplementary motor area (16). Chen et al. studied the inner relationship between the salience network and emotional regions in patients with insomnia and found that these patients have increased functional connectivity between the insula and salience network (17). Furthermore, elevated functional connectivity between the insula and emotional circuit (cingulate cortex, thalamus, and precuneus) has been observed in PI (18). Taken together, these studies suggest that PI can be viewed as a global rather than focal disorder that affects interregional neural coordination of multiple rather than single functional systems.

Human whole-brain networks can be mapped from different modalities of non-invasive neuroimaging techniques such as resting-state fMRI (R-fMRI). R-fMRI is a promising tool for mapping intrinsic brain connectivity networks and has been widely applied to various brain disorders (19). These networks can be further characterized by graph-based approaches by mathematically modeling them as graphs composed of nodes interconnected by edges. With the graph-based approaches, several features are consistently found in healthy brain networks, such as small-worldness (20), modularity (21), and hubs (22). Moreover, accumulating evidence suggests that abnormalities in these configurations are largely responsible for cognitive and behavioral dysfunctions in various brain disorders (23, 24), However, to date, few studies have examined whether and how PI alters whole-brain network organization from a system-level perspective of network segregation and integrity.

In this study, we used graph-based approaches to investigate topological abnormalities of functional brain networks in individuals with PI and to examine clinical correlates of the alterations. Among numerous graph theory-based measures, we exclusively focused on small-world organization because it is one of the most widely used models for human brain network studies (20). The small-world model, which was originally proposed in terms of parameters of clustering coefficient and characteristic path length (25), is an attractive model to characterize brain networks because the combination of high local clustering and short path length supports the two fundamental organizational principles in the brain: functional segregation and functional integration. Subsequently, the small-world theory is expanded based on two biologically more sensible measures: efficiency and cost (26). Compared with conventional clustering coefficient and characteristic path length measures, the combination of efficiency and cost has a number of technical and conceptual advantages since it can (i) represent how efficiently a network exchanges information at local and global levels with a single measure, (ii) examine the economical small-world properties of a network in the sense of providing high global and local efficiency of parallel information processing at low wiring costs, and (iii) deal with disconnected and/or non-sparse graphs (26, 27). To this end, we collected R-fMRI data from 36 patients with PI and 38 age-, sex-, and education-matched healthy controls (HCs). We then constructed individual functional brain networks by calculating interregional functional connectivity of spontaneous blood oxygen level dependent (BOLD) time series signals among 246 regions of interest (ROIs). Next, graph theory-based approaches were used to topologically characterize the resultant networks at global and nodal levels. Finally, PI-related network alterations were statistically inferred using a nonparametric permutation test and correlated with the results of patient neuropsychological assessment.

## Materials and Methods

### Subjects

Patients with PI were recruited from the Department of Neurology at Guangdong No. 2 Provincial People’s Hospital in Guangzhou, China from April 2014 to April 2016. The diagnosis of PI was made according to the Diagnostic and Statistical Manual of Mental Disorders, version 5. The exclusion criteria included (i) insomnia secondary to severe mental diseases (e.g., depression, anxiety, or epilepsy), (ii) other sleep disorders, (iii) history of serious organic disease including significant head trauma or loss of consciousness >30 min, (iv) history of medication treatment for insomnia, (v) history of alcohol, drug, or tobacco abuse, (vi) intense signal on conventional T1- and T2-FLAIR MRI, and (vii) female patients who were pregnant, nursing, or menstruating. We enrolled patients with PI who had all of the following symptoms according to the Pittsburgh Sleep Quality Index (PSQI) (28) and the Insomnia Severity Index (ISI) (29): early awakening, difficulty falling asleep, and difficulty maintaining sleep. In total, 36 patients with PI (12 men; mean age = 38.67 ± 9.53 years) were included in this study. By means of advertisements, we also recruited 38 age-, sex-, and education-matched HCs from the local community (12 men; mean age = 37.79 ± 9.92 years). HCs were included in the study according to the following criteria: (i) good sleep quality and an ISI score <7, (ii) no brain lesions or prior substantial head trauma as verified by conventional T1- or T2-FLAIR MRI, and (iii) no history of psychiatric or neurological diseases. All participants were right handed as assessed using the Edinburgh Handedness Inventory (30). This study was approved by the Ethics Committee of Guangdong No. 2 Provincial People’s Hospital, and all participants provided informed written consent before MR scanning.

### Neuropsychological Assessment

Each participant underwent a series of neuropsychological assessments to evaluate their sleep situation and mental status, including the PSQI, the Epworth Sleepiness Scale (ESS) (31), the ISI, the Self-rating Anxiety Scale (SAS) (32), and the Self-rating Depression Scale (SDS) (32).

### Data Acquisition

All participants were scanned using a 3.0-T Ingenia MRI scanner (Philips Healthcare, The Netherlands) at the Department of Medical Imaging of Guangdong No. 2 Provincial People’s Hospital. During R-fMRI data acquisition, participants were asked to lie quietly with their eyes closed and not think of anything specific or fall asleep while inside the scanner. The detailed acquisition parameters were as follows: repetition time (TR) = 2,000 ms, echo time (TE) = 35 ms, flip angle (FA) = 90°, slice thickness = 3.6 mm with a 0.7-mm gap, matrix = 64 × 64, field of view (FOV) = 230 mm × 230 mm, and 35 transverse planes parallel to the AC–PC line. The R-fMRI scan lasted for 8 min, and a total of 240 volumes were obtained for each participant. In addition, individual high-resolution anatomical images were acquired using a T1-weighted three-dimensional volumetric magnetization-prepared rapid acquisition gradient-echo sequence: 185 axial slices, TR = 8.4 ms, TE = 3.9 ms, FA = 8°, slice thickness = 1.0 mm, no gap, matrix = 256 × 256, and FOV = 256 mm × 256 mm.

### Data Preprocessing

Data preprocessing was performed using the GRETNA toolbox based on the SPM12 package (http://www.fil.ion.ucl.ac.uk/spm/software/spm12/) and included (i) removal of the first 10 volumes to allow for T1 equilibration effects, (ii) realignment to correct for spatial displacements due to head motion, (iii) spatial normalization into the Montreal Neurological Institute space *via* segmentation of structural images, (iv) removal of linear trend, (v) temporal band-pass filtering (0.01–0.08 Hz), and (vi) nuisance regression of white matter signals, cerebrospinal fluid signals, and 24-parameter head-motion profiles (33). Participants with head motion >2 mm or >2° in any direction were excluded. There were no significant differences in the maximum, root mean square, and mean framewise displacement of head motion profiles between groups (all *P* > 0.05). The white matter and cerebrospinal fluid signals were derived by averaging signals within white matter and cerebrospinal fluid masks, respectively, in terms of prior probability maps in SPM12 (threshold = 0.8). We did not regress out global signals because this is a controversial preprocessing step for R-fMRI studies (34).

### Network Construction

We constructed individual functional brain networks in a manner similar to previous studies (35–38). Briefly, we first parceled the cerebrum into 246 ROIs based on a prior brain atlas (39). We then calculated the mean BOLD signal time series for each ROI by averaging the signals across all voxels in that region. Next, the resultant mean time series were correlated with each other to generate a 246 × 246 correlation matrix for each participant. To denoise spurious interregional correlations in the results correlation matrices, we retained only those correlations whose corresponding *P*-values passed through a statistical threshold (*P* < 0.05, Bonferroni-corrected over connections). Such a significance level-based thresholding procedure effectively avoids erroneous evaluations of network topology (40). Finally, negative correlations were excluded due to their ambiguous interpretation (41–43) and detrimental effects on test–retest reliability (44).

### Network Analysis

For the brain networks constructed above, we calculated several graph-based metrics to characterize their topological organization at different levels, including global small-world network efficiency (global efficiency, local efficiency, normalized global efficiency, and normalized local efficiency) and local nodal centrality (nodal efficiency). We briefly explain these metrics below in the context of a weighted network *G* with *N* nodes and *K* edges.

#### Small-World Efficiency

Efficiency is a biologically relevant metric to describe brain networks from the perspective of parallel information propagation and exchange (26, 27) and can be calculated at both global and local levels. Mathematically, global efficiency is defined as:

(1) $$E_{\text{glob}}(G)=\frac{1}{N(N-1)}\sum_{i\neq j\in G}\frac{1}{d_{ij}}$$

where *d_ij_* is the shortest path length between node *i* and node *j* in *G* and is calculated as the smallest sum of edge lengths throughout all possible paths from node *i* and node *j*. The length of an edge was designated as the reciprocal of the edge weight (i.e., correlation coefficient), which can be interpreted as a functional distance that a high correlation coefficient corresponds to a short functional distance. Global efficiency measures the ability of parallel information transmission over the network. The local efficiency of *G* is measured as:

(2) $$E_{\text{loc}}(G)=\frac{1}{N}\sum_{i\in G}E_{\text{glob}}(G_{i})$$

where *E*_glob_*(G_i_)* is the global efficiency of *G_i_*, the subgraph composed of the neighbors of node *i* (i.e., nodes linked directly to node *i*). Local efficiency measures the fault tolerance of the network, indicating the capability of information exchange for each subgraph when the index node is eliminated.

To determine whether brain networks had a small-world organization, local efficiency and global efficiency were normalized *via* dividing them by the corresponding mean derived from 100 random networks that preserved the same number of nodes, edges, and degree distributions as the real brain networks (45–47). Typically, a network with approximately equal global efficiency and larger local efficiency than matched random networks (i.e., normalized global efficiency ~1 and normalized local efficiency >1) is said to be a small-world network (25).

#### Nodal Centrality

We calculated nodal efficiency to capture the centrality of individual nodes in a network. The nodal efficiency of a given node *i* is calculated as (27):

(3) $$e_{i}=\frac{1}{N-1}\sum_{j\neq i\in G}\frac{1}{d_{ij}}.$$

Nodal efficiency measures the ability of information propagation between a node and the remaining nodes in the network. A node with high nodal efficiency indicates high capability of information transmission with other nodes and can therefore be categorized as a hub.

### Statistical Analysis

#### Between-Group Differences in Demographic and Neuropsychological Data

For demographic and clinical variables, between-group differences were examined using two-sample, two-tailed *t*-tests. These variables included age, education, and neuropsychological measurement scores (PSQI, ESS, ISI, SAS, and SDS). In addition, we used a two-tailed chi-square test to determine between-group differences in sex data.

#### Between-Group Differences in Network Organization

Between-group differences in network properties (global efficiency, local efficiency, normalized global efficiency, normalized local efficiency, and nodal efficiency) were determined by nonparametric permutation tests. Briefly, for each network metric, we first calculated the between-group difference in the mean values. An empirical distribution of the difference was then obtained by randomly reallocating all values into two groups and recalculating the mean differences between the two randomized groups (10,000 permutations). The 95th percentile points of the empirical distribution were used as critical values in a one-tailed test of whether the observed group differences could occur by chance. For comparisons of nodal efficiency, the false discovery rate (FDR) procedure was used to correct for multiple comparisons. Given the marginally significant between-group difference in education, we reanalyzed the above comparisons and obtained largely comparable results (data not shown).

#### Between-Group Differences in Functional Connectivity

To examine between-group differences in interregional functional connectivity, a network-based statistic (NBS) method (48) was followed. Briefly, a primary threshold (*P* < 0.05) was applied to the *t*-values (246 × 246 matrix) derived from an edge-by-edge between-group comparison of interregional functional connectivity (two-sample *t*-test). Among the resultant suprathreshold connections, we identified all connected components and recorded their size (i.e., number of links). To estimate the significance of each identified component, a null distribution of the connected component size was empirically derived using a permutation approach (10,000 permutations). For each permutation, all participants were randomly divided into two groups, and the same primary threshold (i.e., *P* < 0.05) was used to filter suprathreshold links in the comparison between the two randomized groups. The size of the maximal connected component among these links was recorded to form the null distribution. Finally, for any connected component of size *M* that was observed in the comparison of the right grouping, the corrected *P* value was determined by calculating the proportion of the 10,000 permutations for which the maximal connected component was larger than *M*. Notably, only connections that were positive in >85% of all participants were included in NBS analysis.

#### Brain–Behavior Relationships

For network metrics that showed significant PI-related alterations, partial correlation analyses were used to assess their relationships with neuropsychological measurements (PSQI, ESS, ISI, SAS, and SDS) and disease duration in the PI group. Effects of age, sex, and education were controlled during the correlation analysis. For the correlation analyses, we did not perform multiple correlation correction given the exploratory nature of this pilot study.

## Results

### Demographic and Clinical Characteristics

Demographic, neuropsychological, and clinical characteristics of participants are shown in Table 1. The HC and PI groups showed no significant between-group differences in age (*P* = 0.699), sex (*P* = 0.872), or education (*P* = 0.054). The average disease duration for participants in the PI group was 28.61 months. As expected, patients with PI had higher PSQI, ISI, SAS, and SDS scores than the controls (*P* < 0.001) (Table 1).

**Table 1:** Demographic, neuropsychological, and clinical characteristics of the participants.

	PI (*n* = 36)	HCs (*n* = 38)	*P* value
Age (years)	38.67 ± 9.53	37.79 ± 9.92	0.699
Gender (M/F)	12/24	12/26	0.872
Education (years)	10.06 ± 3.81	11.66 ± 3.20	0.054
PSQI	11.75 ± 3.78	1.68 ± 1.90	<0.001
ISI	17.28 ± 6.70	1.39 ± 2.40	<0.001
SAS	53.97 ± 10.12	42.45 ± 6.39	<0.001
SDS	52.92 ± 9.25	39.55 ± 10.58	<0.001
ESS	11.00 ± 4.63	–	–
Duration (months)	28.61 ± 43.58	–	–

*PI, primary insomnia; HCs, healthy controls; M, male; F, female; PSQI, Pittsburgh Sleep Quality Index; ISI, Insomnia Severity Index; SAS, Self-rating Anxiety Scale; SDS, Self-rating Depression Scale; ESS, Epworth Sleepiness Scale*.

### Altered Small-World Efficiency in PI

**Figure 1:**
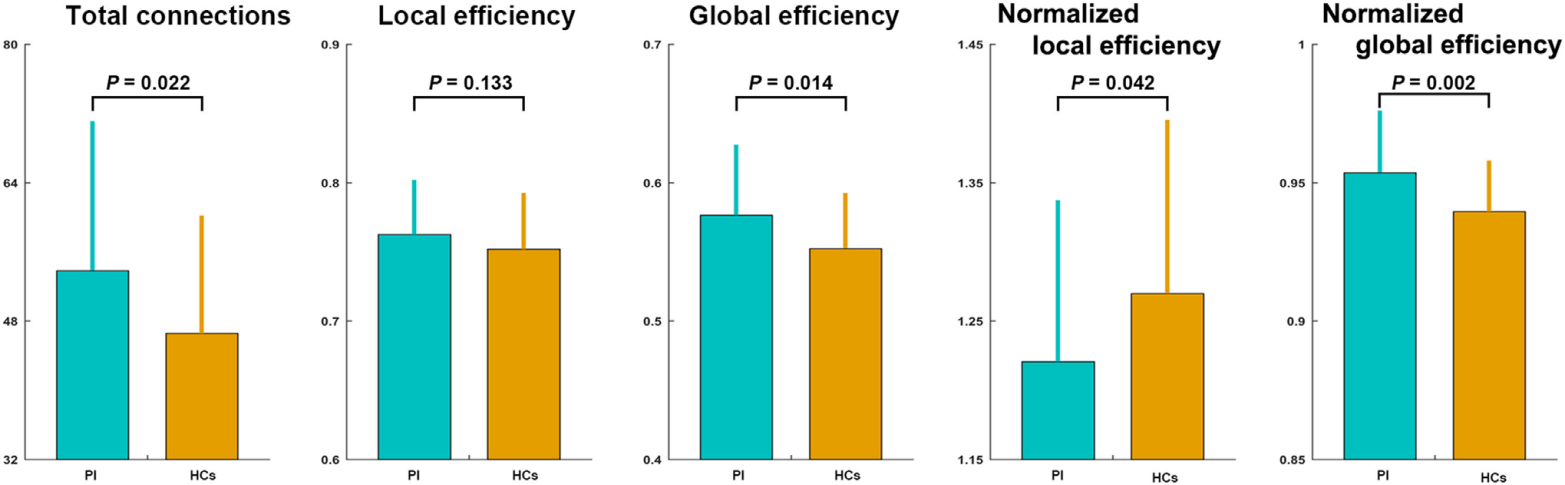
Between-group differences in total number of connections and network efficiency of functional brain networks. Patients with PI had significantly higher total connections (*P* = 0.022), global efficiency (*P* = 0.014), and normalized global efficiency (*P* = 0.002), and lower normalized local efficiency (*P* = 0.042) compared with controls. Error bars denote mean and SD. PI, primary insomnia; HCs, healthy controls.

The mean correlation matrices of the PI and HC groups are shown in Figure 1. We first examined the largest component size of each individual network. We found that networks of 30 HCs and 29 PI patients had no isolated nodes, and networks of all the other participants had one isolated node. Patients with PI had significantly more connections in their whole-brain networks compared with HCs (network density = 0.192 ± 0.056 and 0.222 ± 0.071 for the HC and PI groups, respectively; *P* = 0.022). Network efficiency analysis indicated that the functional brain networks of both groups exhibited small-world organization, as characterized by normalized local efficiency >1 (HC group: 1.270 ± 0.125; PI group: 1.221 ± 0.117) and normalized global efficiency approximately equal to 1 (HC group: 0.940 ± 0.019; PI group: 0.954 ± 0.022). Nevertheless, further statistical comparisons revealed that patients with PI had significantly higher global efficiency (*P* = 0.014) and normalized global efficiency (*P* = 0.002) as well as lower normalized local efficiency (*P* = 0.042) in comparison with HCs (Figure 1).

### Altered Nodal Centrality in PI

**Figure 2:**
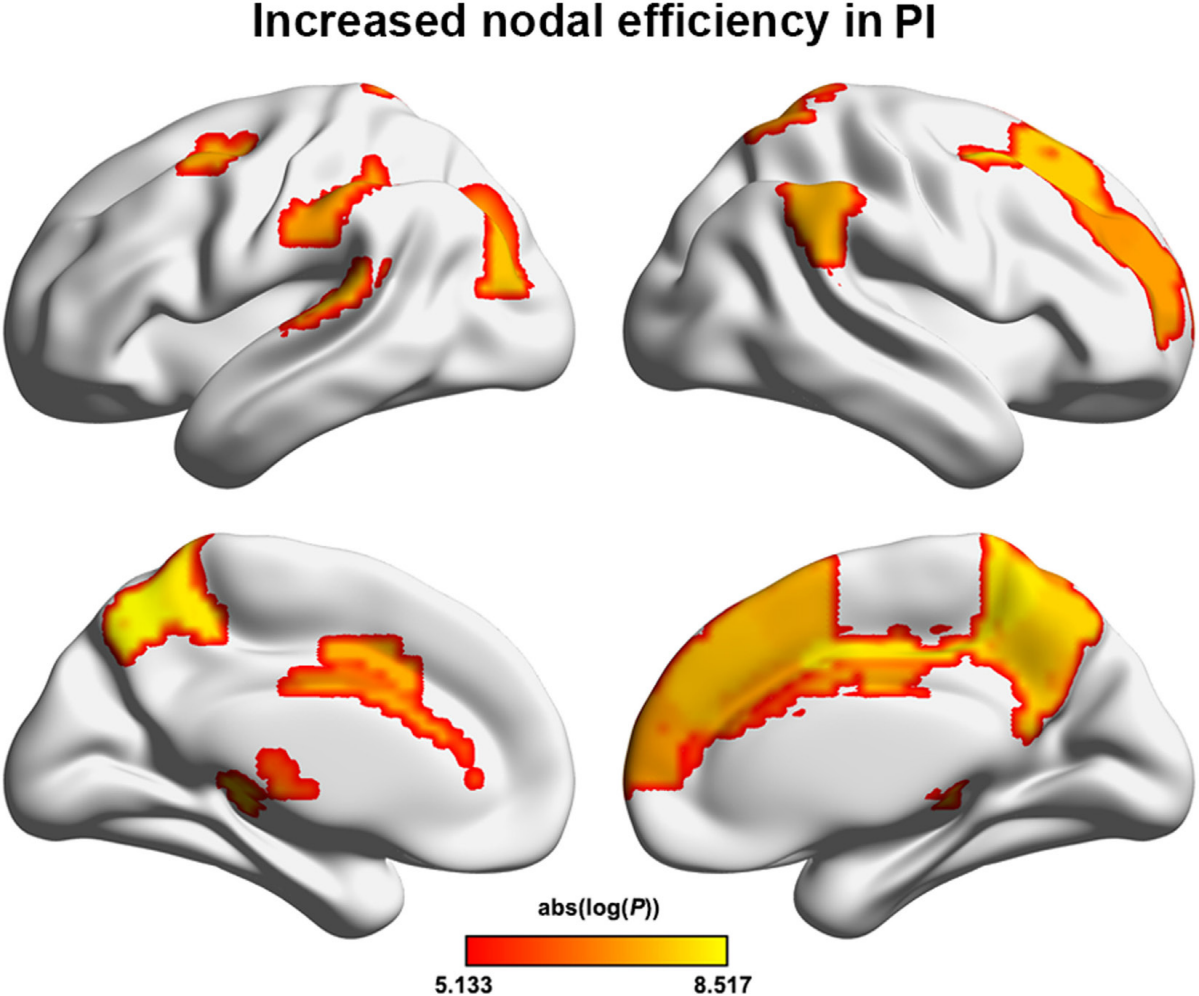
Between-group differences in regional nodal efficiency. Warm color regions show significantly increased nodal efficiency of several prefrontal and parietal regions, the superior temporal gyrus, and the thalamus in patients with PI (*P* < 0.05, corrected). PI, primary insomnia.

The PI group had significantly increased nodal efficiency for 21 regions (*P* < 0.05, FDR corrected) compared with the HC group (Figure 2). These regions predominately encompassed the superior frontal gyrus, middle frontal gyrus, superior temporal gyrus, cingulate gyrus/precuneus, thalamus, superior parietal lobule, and supramarginal gyrus.

### Altered Functional Connectivity in PI

**Figure 3:**
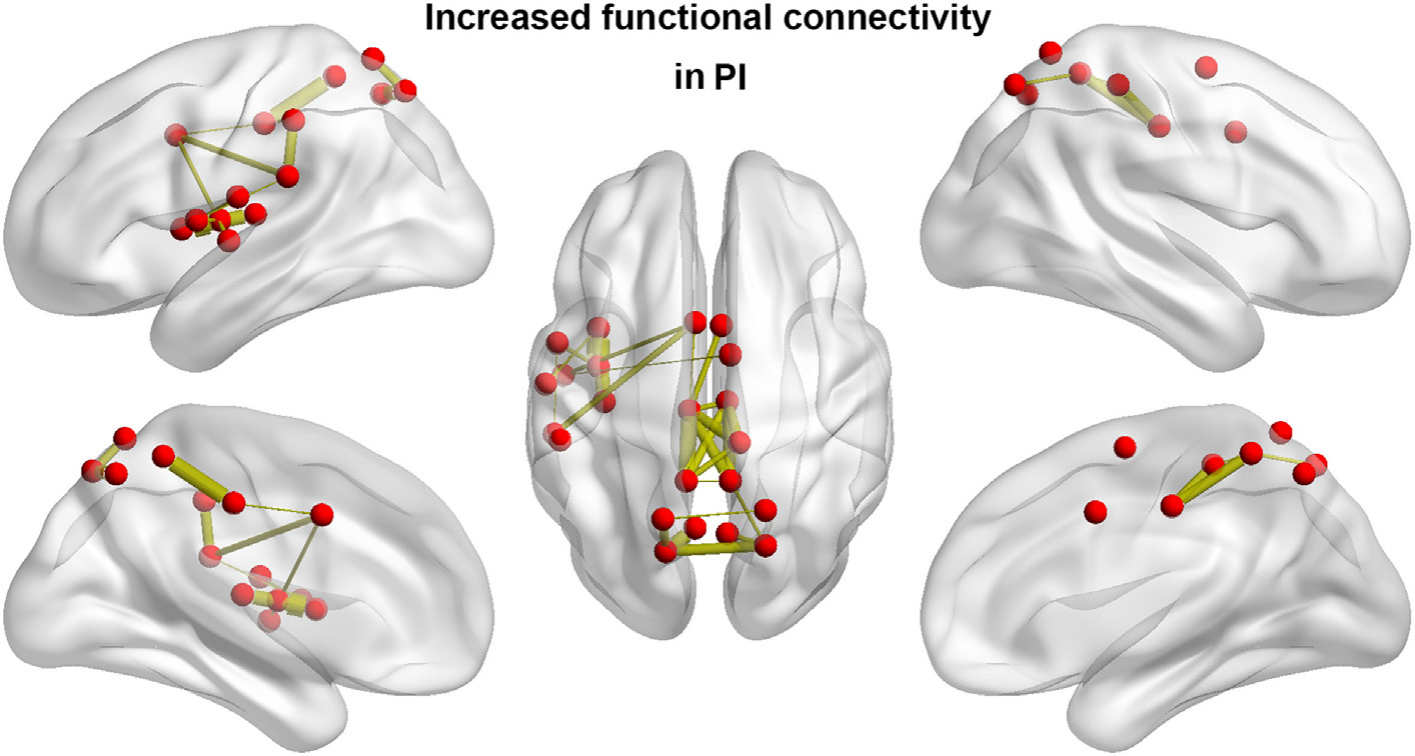
Between-group differences in functional connectivity. Patients with PI showed significantly increased functional connectivity that mainly involved the parietal and prefrontal regions and the insula. Line width is proportional to the significance level of the between-group difference. PI, primary insomnia.

We identified one connected component that exhibited increased functional connectivity among participants with PI as compared with HCs (*P* = 0.044, corrected) (Table 2; Figure 3). The component included 22 nodes and 27 edges and mainly involved the parietal and prefrontal regions and the insula. It is worth mentioning that most of the regions showing increased nodal efficiency, as described earlier, were included in this component. No components showed significantly decreased functional connectivity in a comparison between PI patients and HCs.

**Table 2:** Connections showing increased functional connectivity in the PI patients.

Region A			Region B			
Abbr	Name	MNI	Abbr	Name	MNI	*P* value
SFG_R_7_5	Superior frontal gyrus	[7, −4, 60]	STG_L_6_3	Superior temporal gyrus	[−50, −11, 1]	0.0445
SPL_L_5_1	Superior parietal lobule	[−16, −60, 63]	SPL_R_5_1	Superior parietal lobule	[19, −57, 65]	0.0408
SPL_L_5_1	Superior parietal lobule	[−16, −60, 63]	SPL_L_5_2	Superior parietal lobule	[−15, −71, 52]	0.0087
SPL_L_5_2	Superior parietal lobule	[−15, −71, 52]	SPL_R_5_2	Superior parietal lobule	[19, −69, 54]	0.0059
PrG_L_6_5	Precentral gyrus	[−52, 0, 8]	IPL_L_6_6	Supramarginal gyrus	[−53, −31, 23]	0.0438
IPL_L_6_3	Supramarginal gyrus	[−51, −33, 42]	IPL_L_6_6	Supramarginal gyrus	[−53, −31, 23]	0.0060
SPL_L_5_2	Superior parietal lobule	[−15, −71, 52]	Pcun_L_4_1	Precuneus	[−5, −63, 51]	0.0023
SPL_R_5_2	Superior parietal lobule	[19, −69, 54]	Pcun_R_4_1	Precuneus	[6, −65, 51]	0.0207
PCL_R_2_1	Paracentral lobule	[10, −34, 54]	Pcun_L_4_2	Precuneus	[−8, −47, 57]	0.0151
PCL_R_2_1	Paracentral lobule	[10, −34, 54]	Pcun_R_4_2	Precuneus	[7, −47, 58]	0.0417
SPL_R_5_2	Superior parietal lobule	[19, −69, 54]	Pcun_R_4_2	Precuneus	[7, −47, 58]	0.0282
Pcun_L_4_2	Precuneus	[−8, −47, 57]	Pcun_R_4_2	Precuneus	[7, −47, 58]	0.0442
PrG_L_6_5	Precentral gyrus	[−52, 0, 8]	INS_L_6_5	Rostrodorsal posterior insula	[−38, −8, 8]	0.0242
PoG_L_4_2	Postcentral gyrus	[−56, −14, 16]	INS_L_6_6	Caudoventral anterior insula	[−38, 5, 5]	0.0334
INS_L_6_1	Caudodorsal posterior insula	[−36, −20, 10]	INS_L_6_6	Caudoventral anterior insula	[−38, 5, 5]	0.0021
INS_L_6_5	Rostrodorsal posterior insula	[−38, −8, 8]	INS_L_6_6	Caudoventral anterior insula	[−38, 5, 5]	0.0002
STG_L_6_3	Superior temporal gyrus	[−50, −11, 1]	CG_L_7_5	Cingulate gyrus	[−5, 7, 37]	0.0252
IPL_L_6_6	Supramarginal gyrus	[−53, −31, 23]	CG_L_7_5	Cingulate gyrus	[−5, 7, 37]	0.0154
PCL_R_2_1	Paracentral lobule	[10, −34, 54]	CG_L_7_6	Cingulate gyrus	[−7, −23, 41]	0.0049
Pcun_L_4_2	Precuneus	[−8, −47, 57]	CG_L_7_6	Cingulate gyrus	[−7, −23, 41]	0.0014
Pcun_R_4_2	Precuneus	[7, −47, 58]	CG_L_7_6	Cingulate gyrus	[−7, −23, 41]	0.0046
CG_L_7_5	Cingulate gyrus	[−5, 7, 37]	CG_L_7_6	Cingulate gyrus	[−7, −23, 41]	0.0420
CG_R_7_5	Cingulate gyrus	[4, 6, 38]	CG_L_7_6	Cingulate gyrus	[−7, −23, 41]	0.0170
PCL_R_2_1	Paracentral lobule	[10, −34, 54]	CG_R_7_6	Cingulate gyrus	[6, −20, 40]	0.0062
Pcun_L_4_2	Precuneus	[−8, −47, 57]	CG_R_7_6	Cingulate gyrus	[6, −20, 40]	0.0089
Pcun_R_4_2	Precuneus	[7, −47, 58]	CG_R_7_6	Cingulate gyrus	[6, −20, 40]	0.0048
CG_L_7_6	Cingulate gyrus	[−7, −23, 41]	CG_R_7_6	Cingulate gyrus	[6, −20, 40]	0.0197

### Brain–Behavior Relationship

**Figure 4:**
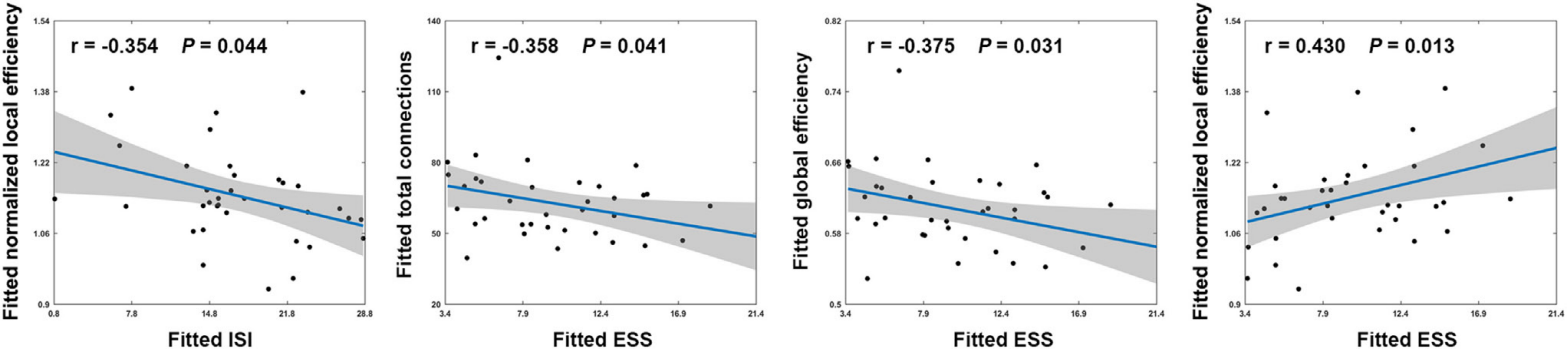
Scatter plots showing significant correlations between network measures and neuropsychological variables in patients with PI. Effects of age, sex, and education were controlled during the correlation analyses. Thus, the fitted values (i.e., the observed network measures and neuropsychological variables minus the estimated effects of age, sex, and education) that are plotted reflect the “true” relationships. ISI, Insomnia Severity Index; ESS, Epworth Sleepiness Scale.

Among patients with PI, ESS scores exhibited significant negative correlations with the total number of edges (*r* = −0.358; *P* = 0.041) and global efficiency (*r* = −0.375; *P* = 0.031) and a significant positive correlation with normalized local efficiency (*r* = 0.430; *P* = 0.013) (Figure 4). In addition, a significant negative correlation was found between ISI scores and normalized local efficiency (*r* = −0.354; *P* = 0.044) (Figure 4). No significant correlations were found between other network measures and neuropsychological variables (all *P* > 0.05).

## Discussion

In this study, we examined the topology of functional brain networks in patients with PI by graph theory-based analysis of R-fMRI. Our results suggest that patients with PI had overly connected functional brain networks as characterized by increased global network efficiency and increased nodal centrality as well as elevated interregional functional connectivity of regions, mainly in the default mode network (DMN) and emotional circuit. Moreover, altered network architecture was related to patient neuropsychological performance. These findings may contribute to a better understanding of the neurobiological mechanisms underlying PI.

The human brain is a complex, interconnected network that continuously integrates information across multiple sensory systems. Numerous studies suggest that its powerful performance originates mainly from a nontrivial topological wiring diagram such as efficient small-world architecture (19, 49, 50). In this study, we found that patients with PI exhibited the increased overall connectivity in their functional brain networks is consistent with previous studies reporting increased functional connectivity and structural connectivity in PI (18, 51, 52). Increased overall connectivity could also explain why increased global efficiency was observed in patients with PI because more connections (i.e., larger network density) might result in more routing paths and therefore more efficient information propagation and exchanges. However, after normalization by matched random networks, global efficiency was still larger in patients with PI than in the controls. This suggests that increased global efficiency of functional brain networks in patients with PI is not entirely due to more connections but instead may reflect an intrinsic alteration in brain wiring patterns. Global efficiency is mainly reflects integrative information processing across brain remote regions that constitutes the basis of cognitive processing (53). Thus, the observed increase of global efficiency implies a hyperactive functional integration of patient brains. This is supported by our findings of increased nodal efficiency and functional connectivity for numerous regions in patients with PI. We discuss the PI-related increases in detail below.

In addition to increased global efficiency, participants with PI had decreased normalized local efficiency which may be explained by impaired functional segregation of patient brains (26). As normal brain function requires an optimal balance between local specialization and global integration, the combination of increased global efficiency and decreased normalized local efficiency indicates a disruption in the normal balance and suggests a tendency toward random network configuration of functional brain networks in patients with PI.

Interestingly, we found that increased overall connectivity and altered network efficiency were related to behavioral disturbances, as indicated by the ESS and ISI scores of patients with PI. ESS is a validated questionnaire that is widely used to assess subjective sleepiness and sleep propensity. Existing evidence suggests that different levels of complaints of daytime sleepiness are broadly experienced by people with insomnia (54). The ISI is a brief instrument that was designed to assess severity of both nighttime and daytime components of insomnia (31). Thus, our results suggest that topological alterations of functional brain networks may account for excessive daytime sleepiness and sleep dysfunction among people with PI.

After examining PI-related network alterations globally, we also investigated PI-related alterations in regional nodal centrality and interregional functional connectivity. At the nodal level, multiple regions that showed increased nodal efficiency were mainly in the DMN (e.g., precuneus, prefrontal cortex, and superior parietal lobule) and emotional circuit (e.g., cingulate cortex, thalamus, and frontal gyrus) in participants with PI. Nodal efficiency measures the extent of information exchange between a given node and all other nodes in a network and therefore reflects the importance or information load of the node (27). Thus, increased nodal efficiency indicates higher information flow of these regions in patient brains. This is consistent with our findings that most of these regions exhibited increased functional connectivity in patients with PI. Interestingly, these regions are largely comparable to those reported to show hypermetabolism in patients with insomnia (12), which is also in agreement with recent findings that functional connectivity is closely coupled with metabolism (55, 56). Future studies are warranted to examine to what extent increased functional connectivity reflects hypermetabolism in PI.

The DMN includes a set of anatomically and functionally interconnected regions that are involved in a wide spectrum of cognitive processing. It is active when individuals are engaged in internally focused tasks such as memory or self-relevant mental processing (57, 58). Using independent component analysis, a previous study found that people with insomnia had increased connectivity of the DMN (59), which is consistent with our findings. Intriguingly, we found that the DMN components affected by PI largely overlapped with the core regions of the mentalizing network (e.g., the medial prefrontal cortex and posterior cingulate cortex/precuneus), which is a subnetwork of the DMN that is typically activated when individuals are engaged in a working memory task (60–62). Clinically, working memory deterioration is the most common symptom of daytime dysfunction in PI (63, 64) and is one of the most apparent and arguably easiest to detect neural markers of PI (65, 66). Based on these findings, we hypothesize that increased nodal efficiency and functional connectivity of DMN regions in PI, particularly the mentalizing network, may reflect a compensatory mechanism of the brain to maintain normal working memory-related processing by adding or establishing new connections.

In addition to the DMN, increases in PI-related nodal efficiency occur in the emotional circuit. The emotional circuit mainly includes the amygdala, prefrontal lobe, thalamus, insular lobe, and cingulate cortex (67). Specifically, the prefrontal lobe and thalamus are central to the perception system and form a channel of prefrontal cortex–thalamus–corpus striatum that cumulatively allows for effective integration and handling of emotional regulation (68). Psychometric studies have demonstrated that emotional hyperarousal may be a primary neural mechanism underlying emotional regulation dysfunction (e.g., anxiety or depression) in patients with PI (69, 70). Similarly, numerous neuroimaging studies have reported overactivity of emotional processing-related brain regions (e.g., prefrontal lobe, thalamus, and insula) in PI (13, 15, 18). Furthermore, electrophysiological studies have found decreased levels of γ-aminobutyric acid in emotion-related regions of PI patients (71). The present findings are consistent with those of previous studies and provide further evidence for the emotional hyperarousal hypothesis from the perspective of functional integration. We hypothesize that increased efficiency in emotional regions may underlie emotional dysfunction frequently observed in PI. Currently, the relationship between insomnia and emotional dysfunction is not fully understood. Previous studies indicate that sleep disturbances have detrimental effects on physical health and are thought to be a risk factor for development and maintenance of mood and anxiety disorders (72–75). Thus, high levels of anxiety and depression are frequently evident in patients with insomnia (76), and high rates of sleep disturbances are observed in patients with anxiety or depressive disorders (77). There are several possible explanations for the interrelationship between anxiety/depression and insomnia. One possibility is that they are simply comorbid, which may be explained by common maintenance mechanisms. Indeed, when we examined relationships between PSQI and ISI scores and scores from SAS and SDS, high positive correlations were found (all *r* > 0.5, *P* < 0.001). A second possibility is that insomnia is epiphenomenal to anxiety and depression or that anxiety and depression are epiphenomenal to insomnia. A third possibility is that anxiety and depression are risk factors for insomnia (78–81). In summary, insomnia and emotional dysfunction are tightly coupled and require further study. Notably, we did not observe significant correlations between increased nodal efficiency and SAS and SDS scores of PI patients, possibly due to the relatively small sample size.

Together, we determined that PI is associated with a hyperactive functional brain connectome as characterized by increased network efficiency and elevated functional connectivity. Currently, the biological mechanism underlying this hyperconnectivity is not fully understood, although it is a common phenomenon in brain disorders (82). Given the high plasticity and compensatory mechanisms of the human brain, one possible interpretation is that the brains of people with PI require ongoing recruitment of available detour paths to maintain normal function by adaptively adjusting regional connectivity profiles in response to pathological attacks and damage caused by the disease (83). More recently, a review indicated that this hyperconnectivity may be optimally expressed by increasing connections through the most central and metabolically efficient regions (84). This is consistent with our findings that increased PI-related efficiency and connectivity were mainly located in the DMN and emotional circuit, which are frequently reported to serve as highly connected hubs in the brain (22). Future studies may provide deeper insights into such hyperconnectivity by combining fMRI with other imaging techniques (e.g., structural and metabolic imaging) and biochemical techniques.

There are several further considerations that merit mention for this pilot study. First, the sample size was relatively small. Therefore, reproducibility of the current findings should be examined in a large cohort of patients. Second, the SDS and SAS scores of patients with PI were still higher than those of participants in the control group. In addition, we found increased functional connectivity of the emotional circle in the PI group. Thus, altered connectivity patterns among participants with PI may not be due to insomnia alone but may also result from secondary mood changes. Further studies are needed to clarify this point. Third, because of the cross-sectional design of this study, we cannot address the temporal relationship between functional brain networks reorganization and progression of PI. Longitudinal studies are needed to illuminate this important issue. Finally, functional brain networks arise from underlying structural pathways (85, 86). Although a recent diffusion tensor imaging study demonstrated abnormalities in several specific neural tracts in PI (87), whole-brain structural networks in PI remain largely unknown.

In summary, from the viewpoint of system-level network separation and integration, this study provides the first evidence for an aberrant functional connectome in PI, which is characterized by increased nodal centrality and interregional functional connectivity in the DMN and emotional circuit. These findings provide novel implications for neural substrates associated with PI.

## Ethics Statement

This study was approved by the Ethics Committee of Guangdong No. 2 Provincial People’s Hospital, and all participants provided their informed written consent before MR scanning.

## Author Contributions

GJ and XM designed experiments. XM, SF, and JF carried out experiments and analyzed experimental results. XM wrote the manuscript. JF assisted with statistical analysis. YW, ML, and TW assisted with carrying out experiments.

## Conflict of Interest Statement

The authors declare that the research was conducted in the absence of any commercial or financial relationships that could be construed as a potential conflict of interest.
